# Molecular Mechanisms of Gas–Ice Interfacial Transport: Size- and Charge-Dependent Fractionation during Bubble Close-off

**DOI:** 10.1021/acsomega.5c08111

**Published:** 2025-11-05

**Authors:** Yoo Soo Yi, Yeongcheol Han

**Affiliations:** † Research Institute of Basic Sciences, 123591 Seoul National University, Seoul 08826, Korea; ‡ Division of Glacier & Earth Sciences, Korea Polar Research Institute, Incheon 21990, Korea

## Abstract

Gas–ice interfacial transport phenomena are essential across diverse cryogenic environments, ranging from gas fractionation in polar glaciers to the preservation of cosmogenic noble gases on icy celestial bodies. Bubble close-off in polar glaciers is a compelling example of the complex gas–ice interactions that challenge the interpretation of paleoclimate records preserved in ice cores. While previous studies have provided valuable insights, the molecular mechanisms governing fractionation, especially those involving both geometric and electronic characteristics, remain incompletely understood. Here, using density functional theory (DFT) calculations, we determine effective permeation energy barriers (*E*_P_) for noble gases (He, Ne, Ar, Kr, and Xe) and molecular gases (N_2_, O_2_, and CO_2_) through a model ice layer. Our results reveal that noble gases largely follow a size-dependent trend, whereas molecular gases deviate from such a simple relationship due to more complex gas–ice interactions resulting from their anisotropic charge distribution. The exponential dependence of permeation rates on *E*_P_ accounts for the observed nonlinear depletion phenomenon. He and Ne, with their smaller sizes and weaker surface adsorption, exhibit higher permeation rates and rapid depletion from closed-off bubbles. Conversely, larger noble gases and molecular gases are preferentially retained due to increased energy barriers. Notably, molecular gases show significantly lower permeation rates than Ne despite comparable effective cross-sectional sizes, owing to stronger adsorption affinity. Chemical hardness, a descriptor reflecting electronic properties, helps reconcile the fractionation patterns observed for both gas types, indicating that interfacial interactions, not molecular size alone, govern transport through ice layers. These findings provide insights into gas preservation in diverse cryogenic environments, which is essential for the fidelity of paleoclimate reconstruction and the rational design of materials for selective transport. Discrepancies with field observations underscore the role of structural heterogeneities, such as grain boundaries, suggesting that bubble close-off fractionation involves additional pathways beyond idealized lattice permeation.

## Graphical abstract

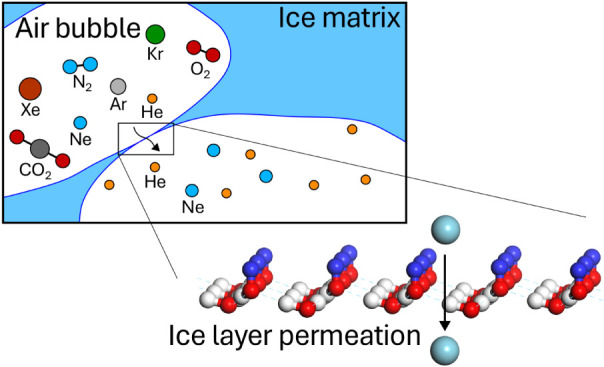

## Introduction

Gas–ice interfacial phenomena play a fundamental role in diverse cryogenic environments, governing the preservation and redistribution of gases entrapped within icy matrices from Earth’s polar regions to icy celestial bodies like Jupiter’s moon Europa. The underlying mechanisms of gas migration are crucial for assessing the fidelity of paleoclimatological and geochronological records preserved in polar glaciers.^1–3^ They also provide insights into the distribution of cosmogenic noble gas isotopes on icy celestial bodies, establishing a potential basis for investigating near-surface geological phenomena through radiometric dating.^4,5^ Hexagonal ice has further been proposed to serve as a temperature-tunable molecular sieve for light gases on the basis of selective permeation.^6^ Despite this broad relevance, a comprehensive molecular-level framework describing these interfacial dynamics has not yet been fully established, making in-depth research essential for both fundamental science and industrial applications.

Among these phenomena, the compositional fractionation of gases during bubble close-off in polar glaciers is a compelling example of complex gas–ice interactions, posing a long-standing challenge to the accurate interpretation of paleoclimate records reconstructed from ice cores. Trapped deep within the stratified ice of polar glaciers, gaseous remnants of ancient climates serve as time capsules of Earth’s atmospheric history, offering an unparalleled window into climate variability, geological activities, and anthropogenic impacts.^2,3,7–12^ One critical process affecting gas records occurs during bubble close-off, in which firn air becomes sealed within newly formed enclosed air pockets at depths down to ∼100 m.^13–15^ Notably, previous studies have revealed considerable compositional differences between newly formed enclosed air bubbles and the surrounding open pores, particularly the depletion of smaller gases from bubbles and their enrichment in residual pore spaces.^13–15^ This trend has been attributed to size-dependent permeation through ice matrices.^4,16–19^ However, the abrupt and nonlinear decrease in depletion beyond a certain molecular size suggests the need for a more nuanced understanding that incorporates unexplored physicochemical processes. Previous studies, including our theoretical investigation of gas migration at the gas–ice interface, have examined various aspects of layer-specific gas transport in glacial ice, from intermediate depths where bubbles appear stable to deeper zones where clathrate hydrates form.^4,13–15,17,19–36^ These complexities underscore the need for a comprehensive molecular-level framework to understand broader ice-related interfacial phenomena.

Recent advances in theoretical simulations have enabled a more comprehensive investigation of interfacial gas transport. A previous theoretical study revealed size-dependent trends in the energy barriers for both dissolution and subsequent molecular diffusion of noble gases from isolated air bubbles into the surrounding ice matrix.^4^ The correlation between dissolution energy barriers and solubility, interpreted within the framework of transition state theory (see refs 37–39 for details), suggests that gas fractionation at the gas–ice interface may be governed by size-sensitive dynamics characterized by an Arrhenius-type relationship. This relationship offers a plausible explanation for the nonlinear depletion of larger gases observed in previous studies.^4,13–15^ Although these findings contribute to our understanding, further investigation into how gases permeate the thin ice layers enclosing newly formed bubbles is required to fully understand gas migration during bubble close-off.

Furthermore, electrostatic interactions at the gas–ice interface remain largely unexplored. Although the precise structure of these thin ice layers is not well characterized (details in Methods), the potential presence of reactive sites on the ice surface suggests that gases characterized by an anisotropic charge distribution and significant polarizability, such as O_2_, N_2_, and CO_2_, may exhibit distinct interfacial transport dynamics, as demonstrated in previous studies of molecular adsorption onto ice surfaces.^40–46^ To address these unresolved aspects, we investigated gas permeation through an idealized ice layer model based on the conceptual framework proposed in previous studies, thereby advancing our understanding of gas fractionation phenomena in natural ice systems. While empirical potential-based classical molecular dynamics can capture bulk dynamics in H_2_O systems, such methods often exhibit diminished accuracy for configurations where empirical validation remains challenging, particularly transition states during bulk ice lattice diffusion where intermolecular interactions increase rapidly (see ref 16 for details). Therefore, a comprehensive molecular-level investigation of gas fractionation during bubble close-off was performed in this study using density functional theory (DFT) calculations. Through transition state searching and nudged elastic band (NEB) calculations, we determined the minimum energy paths (MEPs) for various gases, including noble gases (He, Ne, Ar, Kr, and Xe) and molecular gases (N_2_, O_2_, and CO_2_), permeating the thin ice layers (see Figure 1). Our analysis offers new molecular-level insights into the underlying mechanisms during bubble close-off and reveals the intricate interplay of both size- and charge-dependent processes at the gas–ice interface. These findings will help to establish more comprehensive models that transcend the prevailing paradigm for gas fractionation phenomena in natural polar glaciers, providing a foundation for future research.

**Figure 1:**
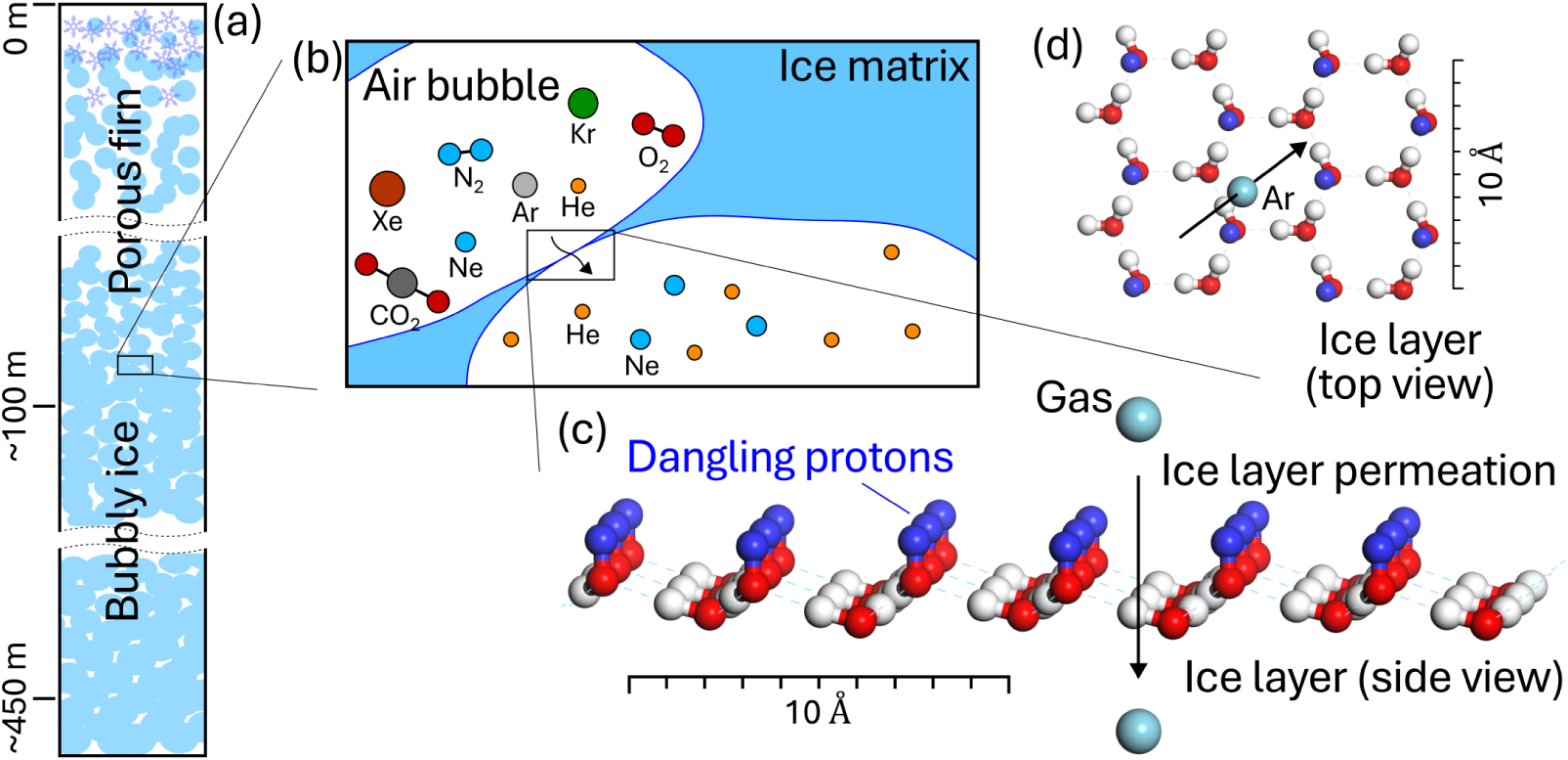
Schematic illustration of gas fractionation near the depths where bubble close-off occurs. (a) Stratified layers in polar glaciers, showing firnification and the firn-to-ice transition in porous firn and bubbly ice in deeper regions. (b) Gas fractionation through the thin ice layer during bubble close-off, showing depletion of smaller gases from enclosed air bubbles and enrichment in residual firn air. (c–d) Ice layer model consisting of a single bilayer of water molecules cleaved along the basal (0001) plane used to examine the gas permeation process. In (c) and (d), upward-facing dangling protons are highlighted in blue.

## Methods

### Structural Model

To investigate gas fractionation during bubble close-off in glacial ice, we prepared a membrane-like ice structure as an analogue of the thin ice layers that form during the early stages of bubble close-off (Figure 1). This structure consists of a single bilayer of water molecules cleaved from ice-Ih along the (0001) basal plane, which is a naturally preferred direction known to produce a surface with ordered OH groups and upward-facing dangling protons. While natural ice-Ih typically contains structural defects, such as ionic vacancies and molecular disorder, we adopted a defect-free structure (known as ice-XI) to simplify the model.^47–49^ In the ice layer model, a vacuum spacing of ∼52.7 Å and a surface area of ∼13.2 × 22.8 Å^2^, containing a total of 36 water molecules, were employed. In our previous study, we demonstrated that spurious interactions with periodic images in both the vertical and lateral directions are effectively minimized by using models with comparable surface areas and vacuum spaces (details in ref 46).

We designed our ice layer model based on the relatively well-characterized cleaved surface of bulk ice.^50–53^ While the precise structure of thin ice layers is yet to be fully established, previous studies have provided insights into their plausible structures.^54–60^ Under ambient pressure, these layers exhibit a hexagonal network structure composed of alternating water molecules with out-of-plane dangling protons and in-plane hydrogen bonds.^54,58^ The orientation of these out-of-plane dangling protons strongly influences the surface reactivity, particularly for molecular gases with characteristic charge distributions. However, the subtle stability variations among different possible arrangements of these dangling protons do not markedly affect the overall structural stability of thin ice layers, making it challenging to define a definitive structure.^57^ Despite these structural uncertainties, the gradual thickening of thin ice layers during the firn-to-ice transition may make their surfaces more comparable to those of bulk ice. Previous spectroscopic and theoretical studies have suggested that well-ordered domains with upward-facing dangling protons likely persist on the cleaved ice surface even at higher temperatures (but below the melting point) or under quasi-liquid conditions, primarily because of coherent vibrational coupling among the surface water molecules.^61–68^ Notably, other recent studies on thin ice layers deposited on various substrates have revealed structural complexities beyond those observed in an ideally cleaved ice-Ih surface, such as mixed nanodomains with hexagonal and cubic symmetries and an alternating arrangement of dangling protons and dangling oxygen atoms.^69–72^ While the arrangement of dangling protons on cleaved ice surfaces remains under debate, such structural details have minimal influence on the stability of thin ice layers, as discussed above.^57^ Considering this, we employed a simplified ice layer model with fully ordered upward-facing dangling protons. This model, though simplified, effectively captures the fundamental principles governing gas permeation through the ice layers.

### DFT Calculations

We employed DFT methods using the Cambridge Serial Total Energy Package (CASTEP), a plane-wave pseudopotential DFT program, to investigate gas fractionation through thin ice layers during bubble close-off.^73^ The calculations were conducted using on-the-fly generated ultrasoft pseudopotentials and the Perdew–Burke–Ernzerhof (PBE) exchange-correlation functional, which was enhanced with Grimme’s D4 dispersion correction (PBE-D4).^73–76^ Notably, the PBE-D4 method yields robust and accurate results comparable to those of other high-level methods, such as the dispersion-corrected strongly constrained and appropriately normed (SCAN) and revised van der Waals density functional 2 (rev-vdW-DF2) methods.^75,76^ Electronic structure calculations were carried out with a planewave cutoff energy of 600 eV and a k-point sampling grid of 1 × 1 × 1. The self-consistent field (SCF) energy convergence threshold was set to 1.0 × 10^–6^ eV/atom. For systems containing O_2_ molecules, calculations were performed considering the triplet ground state of O_2_. Prior to transition state identification, structure optimizations were performed using the two-point steepest descent (TPSD) algorithm with convergence criteria of 1.0 × 10^–5^ eV/atom for total energy, 0.03 eV/Å for force, 0.05 GPa for stress, and 0.001 Å for atomic displacement.^77^ The transition states for gas permeation were then identified using the complete linear synchronous transit and quadratic synchronous transit (LST/QST) method with a root-mean-square (RMS) force convergence criterion of 0.05 eV/Å.^78^ The MEP was then refined through nudged elastic band (NEB) calculations using convergence thresholds of 1.0 × 10^–5^ eV/atom for total energy, 0.03 eV/Å for force, and 0.001 Å for displacement, with a spring constant of 0.1 eV/Å^2^.^79^ During these calculations, gas molecules were positioned at least 10 Å from the ice layer in both the initial and final states of the overall permeation process to minimize gas–ice interactions, ensuring that the calculated MEP captures only the essential permeation barrier. The parameters for DFT calculations and ice layer dimensions were determined based on convergence tests performed in our previous studies using bulk ice lattice and cleaved ice surface models.^4,16,46^ The spring constant for NEB calculations is a practical parameter that requires system-specific adjustment. While this parameter influences the distribution of images along the reaction path, it does not significantly affect the final MEP results in our case (when using 20 NEB images, no significant differences in transition states were observed with spring constants ranging from 0.05 to 0.2 eV/Å^2^). In addition, the electrostatic potential (ESP) surfaces of molecular gases were calculated using the semiempirical linear combination of atomic orbitals (LCAO) method with the PM6 parameterization of the neglect diatomic differential overlap (NDDO) Hamiltonian, as implemented in the VAMP module of Materials Studio.^80–82^ Considering their respective ground-state spin multiplicities, we employed the restricted Hartree–Fock (RHF) formalism for N_2_ and CO_2_ (closed-shell singlet species) and the unrestricted Hartree–Fock (UHF) formalism for O_2_ (open-shell triplet ground state).

## Results and Discussion

### Energy Profiles for Gas Permeation through Ice Layer

To elucidate how gas fractionation occurs at the gas–ice interface, we investigated the energy variations during the permeation of noble gases and molecular gases through the thin ice layer, as illustrated in Figure 1. As shown in Figure 2, gas permeation through the ice layer occurs via a multistep process. A gas molecule approaches the ice surface (initial state, IS) and becomes weakly adsorbed at the gas–ice interface (intermediate state 1, IM1). To penetrate the ice layer, the gas molecule must overcome the highest energy point (transition state, TS) in the overall energy profile. This IM1-to-TS transition constitutes the rate-limiting step of the entire permeation process. While the ice layer structure remains largely undisturbed along the IS-to-IM1 path, significant perturbations in the hydrogen bonding network become apparent at the TS, where the gas molecule occupies the center of the hexagonal ring. The gas molecule subsequently reaches another weakly adsorbed state on the opposite side (intermediate state 2, IM2) before fully desorbing (final state, FS).

**Figure 2:**
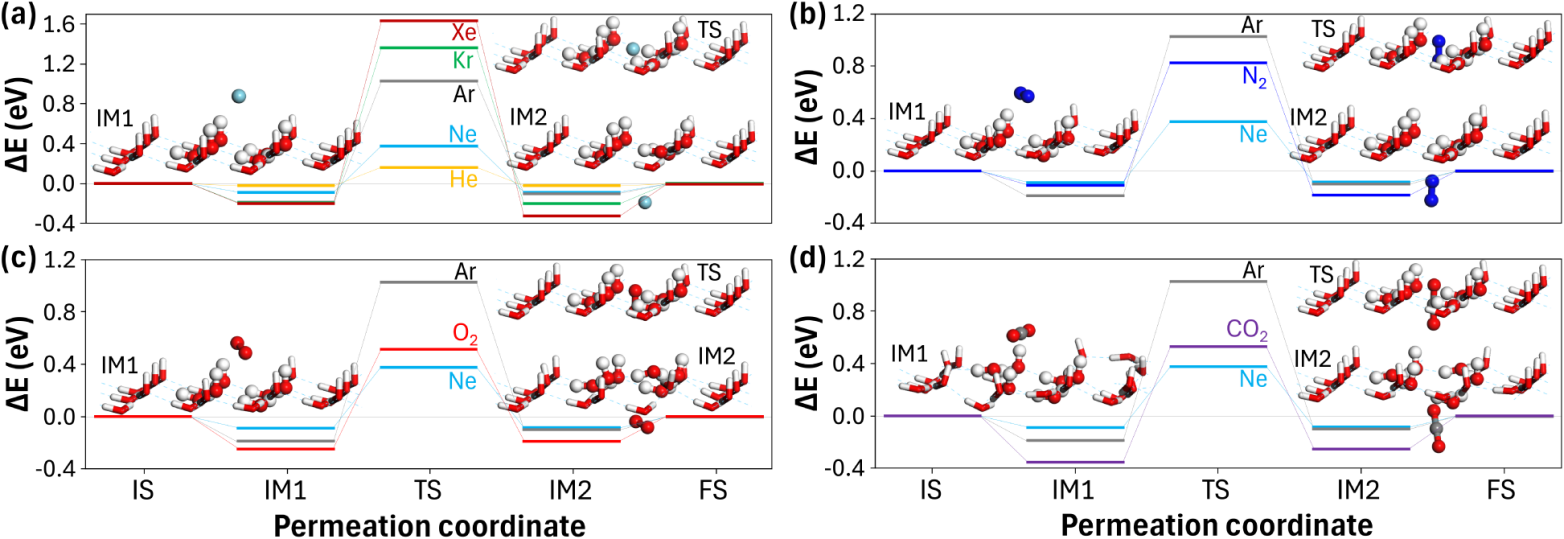
Energy profiles for gas permeation through the ice layer. Energy differences (Δ*E*) relative to the initial state (IS) for (a) noble gases (He, Ne, Ar, Kr, and Xe) and (b–d) molecular gases (N_2_, O_2_, and CO_2_) along the permeation pathway: initial state (IS), intermediate state 1 (IM1), transition state (TS), intermediate state 2 (IM2), and final state (FS). For comparison, Ne and Ar results are included in panels (b–d). Representative molecular configurations for IM1, TS, and IM2 are shown in each panel.

Figure 2a presents the energy variations (Δ*E*) of noble gases, demonstrating temporary trapping at both intermediate states (Δ*E* < 0 at IM1 and IM2) through nonbonded adsorption to the ice surface. The strength of this trapping appears to increase with an increase in atomic size. Noble gases interact with the electrostatically polarized ice surface, characterized by dangling protons (Figure 1), through induced dipole–dipole interactions stemming from their instantaneous polarization. Molecular gases (N_2_, O_2_, and CO_2_) also exhibit temporary trapping on the ice surface, with negative Δ*E* values observed at IM1 and IM2 (Figure 2b–d). These molecular gases tend to align parallel to the ice surface when adsorbed (IM1) but reorient vertically when penetrating through the hexagonal ring at TS, as illustrated in Figure 2b–d (see also refs 4,16 for the diffusion of such molecular gases in bulk ice). On the opposite side of the ice layer (IM2), steric repulsion between the penetrating gases and the downward-facing oxygen atoms of the ice layer perturbs the local hydrogen bonding network. These energy profiles reveal the intricate nature of gas fractionation and demonstrate nonbonded adsorption at the gas–ice interface during permeation through the ice layer. Building upon these observations, we proceeded to elucidate the molecule-specific properties that govern nonbonded interfacial interactions to better understand the permeation dynamics and fractionation behavior.

### Electrostatic Properties of Molecular Gases

To understand the variations in the permeation behavior observed in Figure 2, we now examine the molecular-level origins of these differences. Figure 3 presents the ESP surfaces of the molecular gases (N_2_, O_2_, and CO_2_), revealing distinctive electrostatic properties stemming from their bonding characteristics and molecular orbital configurations.^83,84^ For N_2_, the strong triple bond localizes the electron density between the nitrogen atoms, resulting in a distinct region of negative ESP along the bond axis.^85–87^ In contrast, the ESP surface of paramagnetic O_2_ shows more pronounced negative regions near the oxygen atoms (Figure 3b). This behavior stems from the presence of two unpaired electrons occupying antibonding π* orbitals oriented perpendicular to the bond axis with a nodal plane between the atoms.^88,89^ The CO_2_ molecule has a distinctly anisotropic ESP surface, with a positive region around the carbon atom and negative regions around the oxygen atoms (Figure 3c). This charge separation results primarily from the high electronegativity of the oxygen atoms, although it is tempered by π-electron redistribution from oxygen lone pairs.^85,90^ These distinct electrostatic properties provide the molecular-level basis for the differences in gas–ice interactions and their consequent influence on the permeation behavior through the ice layer (see Figure 4 and related discussion).

**Figure 3:**
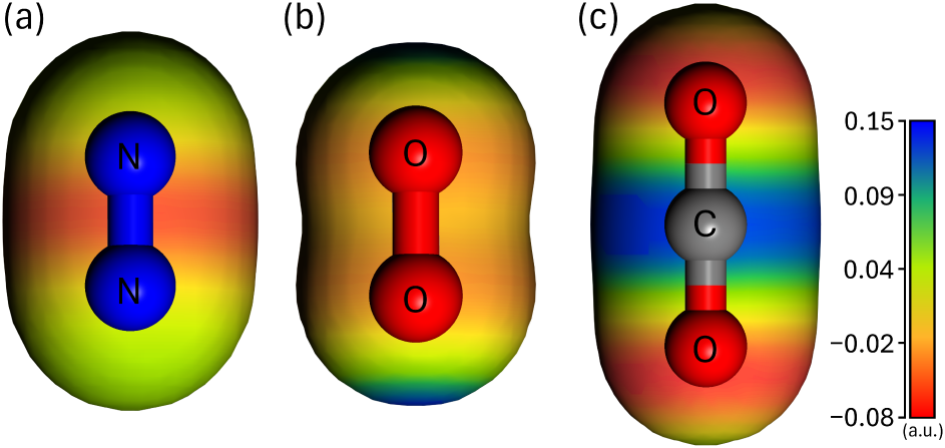
Electrostatic characteristics of molecular gases. Electrostatic potential (ESP) surfaces of (a) N_2_, (b) O_2_, and (c) CO_2_ molecules. ESPs are projected onto the electron density isosurfaces of 0.02 e/Å^3^ for each molecule, mapped from −0.08 (red, negatively charged electron-rich region) to 0.15 a.u. (blue, positively charged electron-deficient region).

**Figure 4:**
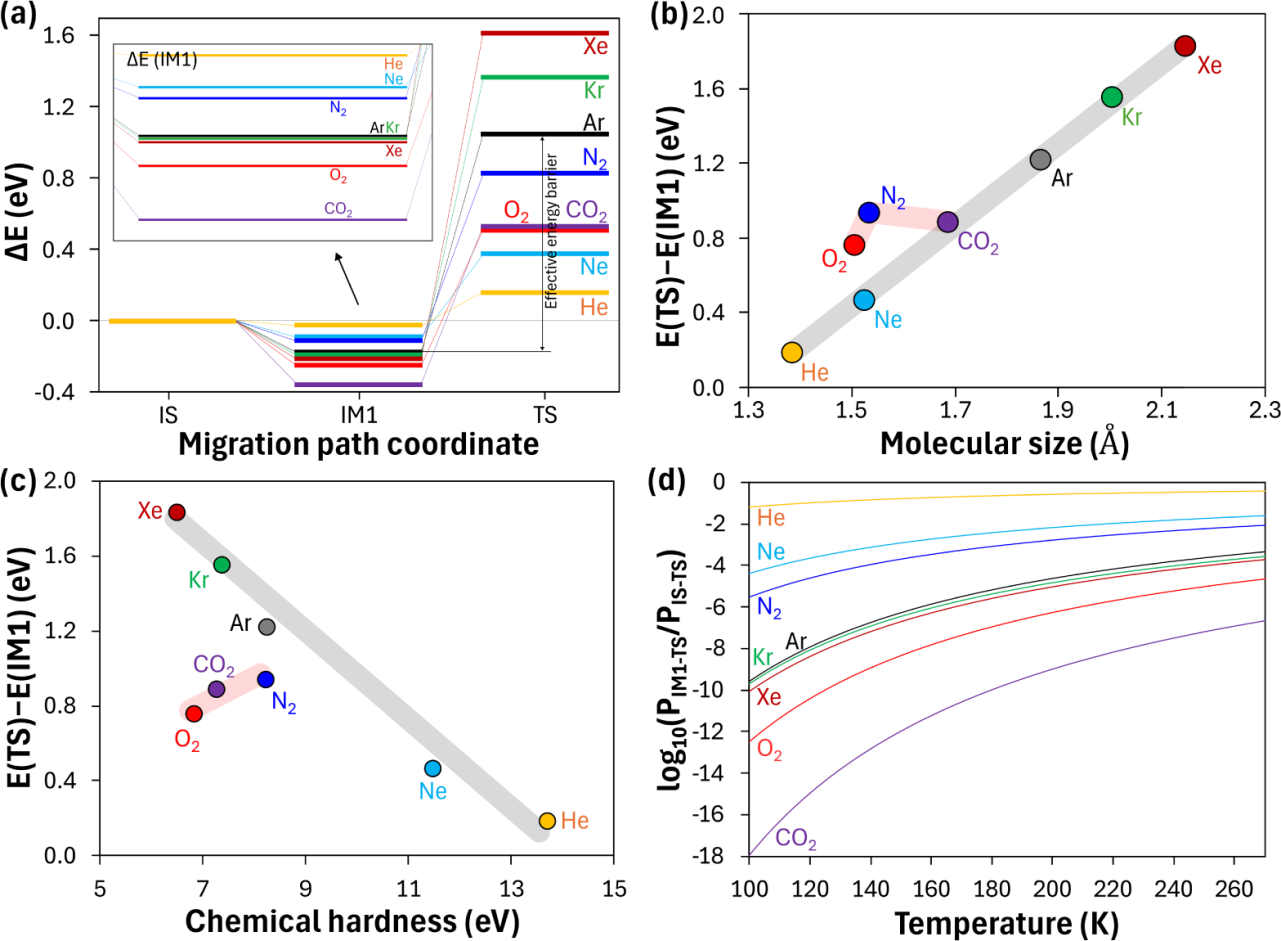
Energetics of gas permeation through the ice layer. (a) Energy differences (Δ*E*) relative to the initial state (IS) for noble gases (He, Ne, Ar, Kr, and Xe) and molecular gases (N_2_, O_2_, and CO_2_), along the initial state (IS), intermediate state 1 (IM1), and transition state (TS). (b–c) Effective energy barriers, defined as *E*(TS) – *E*(IM1), for noble gases (gray lines) and molecular gases (red lines), plotted against (b) molecular size (effective cross-sectional vdW radius during permeation) and (c) chemical hardness (see Table S1). (d) Logarithmic permeability ratio, defined as log_10_(*P*_IM1–TS_/*P*_IS–TS_), as a function of temperature from 100 to 270 K.

### Energy Profiles for IS–IM1–TS Transition

As shown in Figure 2, both noble gases and molecular gases experience transient adsorption at the gas–ice interface. To accurately capture the permeation dynamics, we should account for the energy required for the IM1-to-TS transition, which incorporates the influence of nonbonded adsorption, defined here as the effective permeation energy barrier *E*(TS) −*E*(IM1). Figure 4a presents the energy profiles for each gas species along the IS–IM1–TS path, offering detailed insights into the effective permeation energy barriers. For noble gases, Δ*E*(TS) increases systematically with increasing atomic size, ranging from 0.16 eV for He to 1.63 eV for Xe (He < Ne < Ar < Kr < Xe), which is consistent with greater steric hindrance for larger atoms. Despite being chemically inert, noble gases exhibit negative Δ*E*(IM1) values (inset, Figure 4a), indicating energetically favorable trapping at the gas–ice interface. From He to Ar, Δ*E*(IM1) decreases due to the increased polarizability with increasing atomic size, indicating significantly stronger adsorption. Beyond Ar, this trend becomes negligible, as attractive forces are counterbalanced by increased equilibrium distances and greater Pauli repulsion, resulting in nearly constant Δ*E*(IM1) values (He < Ne < Ar ∼ Kr ∼ Xe).^75,76,91^

Molecular gases (N_2_, O_2_, and CO_2_) exhibit more complex behavior (Figure 4a) than noble gases because of their distinctive electronic properties (Figure 3). At the ice layer surface, the Δ*E*(IM1) of N_2_ (−0.11 eV) shows weak adsorption similar to Ne due to its chemical inertness (Figure 3a), while O_2_ (−0.25 eV) and CO_2_ (−0.36 eV) exhibit stronger surface interactions through their electronegative oxygen atoms (Figure 3b and c). Notably, despite its weak adsorption, N_2_ shows the highest Δ*E*(TS) (0.83 eV) compared to O_2_ (0.51 eV) and CO_2_ (0.53 eV), suggesting that steric factors dominate its permeation behavior. In contrast, the O_2_ and CO_2_ molecules benefit from their ability to be incorporated into the surrounding hydrogen-bonding network, facilitating more favorable transition states.

### Effective Permeation Energy Barrier vs Molecular Size

Figure 4b presents the effective permeation energy barrier, *E*(TS) – *E*(IM1), with respect to the molecular size (with energy values derived from Figure 2). The van der Waals (vdW) radius was employed for noble gases, whereas for molecular gases, the vdW radius of their largest atom was used to capture the effective cross-sectional dimension during permeation (see also Figure 2 for the molecular orientation of gases). The *E*(TS) – *E*(IM1) of noble gases steadily increases with increasing atomic size, from 0.18 eV (He) to 1.83 eV (Xe). This trend stems from the combined contributions of both *E*(TS) and *E*(IM1): *E*(TS) increases with increasing atomic size due to greater steric hindrance during permeation, and *E*(IM1) becomes more negative as a result of the higher polarizability of larger noble gases (Figure 4a).

In contrast, molecular gases deviate from the straightforward relationship established for noble gases, as molecule-specific electronic properties significantly influence their interactions with out-of-plane protons of the ice layer. For instance, N_2_ exhibits a substantially higher *E*(TS) – *E*(IM1) than Ne, despite their similar cross-sectional dimensions. Given the chemical inertness of N_2_ (Figure 3a), the higher barrier arises primarily from steric hindrance due to its diatomic structure, while its Δ*E*(IM1) remains modest and comparable to Ne (Figure 4a). Conversely, O_2_ and CO_2_ show lower Δ*E*(TS) values than N_2_ (Figure 4a) through favorable transition states facilitated by their electronegative terminal oxygen atoms (Figure 3b and c); however, their stronger surface adsorption, reflected in more negative Δ*E*(IM1) values (Figure 4a), results in *E*(TS) – *E*(IM1) values comparable to N_2_ (Figure 4b).

### Effective Permeation Energy Barrier vs Chemical Hardness

Figure 4c shows the relationship between *E*(TS) – *E*(IM1) and chemical hardness. Chemical hardness (η), which is a quantitative measure of resistance to electron density deformation under perturbative influences, was calculated as (*I* – *A*)/2, where *I* and *A* indicate the ionization potential and electron affinity, respectively (see Table S1 for each value of DFT-based chemical hardness).^92–94^ For noble gases, as atomic size increases, the greater number of electrons enhances nuclear shielding, leading to higher atomic polarizability, which in turn results in lower chemical hardness. For molecular gases, chemical hardness exhibits more complex trends due to factors such as bond characteristics, electronegativity differences, and molecular orbital configurations that collectively influence their electronic properties (Figure 2).

Noble gases exhibit a clear negative correlation between the effective permeation energy barrier *E*(TS) – *E*(IM1) and chemical hardness (d*E*/dη < 0). This trend is attributed to the fact that a lower chemical hardness corresponds to both increased polarizability (strengthening surface adsorption) and larger atomic size (increasing steric hindrance). In contrast, molecular gases show a positive correlation (d*E*/dη > 0) due to their distinct electronic and bonding characteristics. Molecular gases with lower chemical hardness (O_2_ and CO_2_) exhibit lower *E*(TS) – *E*(IM1) values despite their stronger surface adsorption, because of their favorable transition states facilitated by hydrogen bonding with surrounding out-of-plane dangling protons. In contrast, N_2_, with a higher chemical hardness, shows the opposite trend, with weak adsorption yet high permeation barriers (as detailed in Figures 3 and 4a). The trends observed for molecular gases in Figure 4b and c are specific to the gas species investigated in this study. Other molecular gases may exhibit different patterns based on their molecular sizes and physisorption interactions with ice surfaces.

### Contribution of *E*(IM1) to Permeability

Figure 4d presents the temperature-dependent variations in the permeability ratios for the IM1-to-TS and IS-to-TS transitions (*P*_IM1–TS_/*P*_IS–TS_) over the temperature range of 100–270 K. This Arrhenius-based analysis shows how Δ*E*(IM1) contributes to the overall permeation behavior.^4,16,37–39^ Both pathways share the same TS with only slight differences in the initial geometry between IS and IM1, since the weak gas–ice interactions do not significantly perturb the ice layer structure at IM1. Thus, their preexponential factors, which are determined by the attempt frequency and proportional coefficient, are assumed to be nearly identical (details in refs 4, 16, 37–39 and Section 1 of the SI). Consequently, the (*P*_IM1–TS_/*P*_IS–TS_) ratio is predominantly governed by the energy difference between IS and IM1 as follows: (*P*_IM1–TS_/*P*_IS–TS_) ∼ exp­(−Δ*E*_p_/*k*_B_ *T*), where Δ*E*_p_ = *E*(IS) – *E*(IM1). Thus, even small changes in *E*(IM1) can lead to dramatic differences in permeability.

The log_10_(*P*_IM1–TS_/*P*_IS–TS_) values presented in Figure 4d show distinct gas-specific temperature dependences. Given the direct correlation of this ratio with the strength of gas–ice interactions, represented by Δ*E*(IM1), gases with stronger interactions, such as CO_2_, O_2_, and larger noble gases, exhibit more pronounced deviations from zero. For instance, the differences in *E*(IM1) among N_2_ (−0.11 eV), O_2_ (−0.25 eV), and CO_2_ (−0.36 eV) shown in Figure 4a yield substantial differences in permeability spanning 12 orders of magnitude at 100 K (2.81 × 10^–6^, 3.15 × 10^–13^, and 1.06 × 10^–18^, respectively). With increasing temperature, the permeability ratios for all gases systematically increase, indicating that the contribution of gas–ice interactions decreases due to increased molecular mobility at elevated temperatures. This trend is particularly pronounced for gases with low chemical hardness, which strongly interact with the ice layer. For example, CO_2_, with the lowest *E*(IM1), shows the most dramatic response, with its *P*_IM1–TS_/*P*_IS–TS_ ratio increasing by 11 orders of magnitude (from 1.06 × 10^–18^ to 2.20 × 10^–7^) as temperature increases from 100 to 270 K. These results thus demonstrate the substantial influence of gas–ice interfacial interactions on the overall permeation dynamics.

### Gas Fractionation Behavior during Bubble Close-off

The size- and charge-dependent effective energy barriers for the permeation of noble gases and molecular gases through the ice layer, as presented in Figure 4 allow us to estimate the relative fractions of gases retained within enclosed air bubbles during bubble close-off in polar glaciers. To quantitatively model this compositional fractionation, we employed first-order reaction kinetics, expressed as *N*(*t*)/*N*_0_ = exp­(−*kt*), where *N*_0_ is the initial number of gas molecules, *k* is the permeation rate constant, and *N*(*t*) is the number of gas molecules remaining at time *t* (see Section 1 of the SI and refs 4, 37–39 for details of this analysis). Since the permeation rate constant (*k*) follows an Arrhenius relationship, *k* ∝ exp­(−*E*_P_/*k*_B_ *T*), the effective permeation energy barrier (*E*_P_) becomes the most dominant factor governing this process. This exponential dependence on energy barriers outweighs the linear effects of other parameters (geometric factors and uncertainties in attempt frequency), ensuring robust conclusions about relative fractionation patterns despite numerical approximations. Given the uncertainty in the time scale of bubble close-off fractionation, we employ a dimensionless time scale normalized to the characteristic time when Ne concentration reaches 60% of its initial value, enabling direct comparison of relative remaining fractions among gas species. Figure 5a presents the remaining fractions of gases as a function of molecular size. Our results for noble gases are in broad agreement with those of previous studies, capturing the characteristic nonlinear depletion trend: larger noble gases (Ar, Kr, and Xe) show negligible depletion, whereas smaller noble gases (He and Ne) experience more pronounced depletion.^15^ Despite minor quantitative discrepancies, particularly for He and Ne, the overall behavior remains consistent with earlier observations.

**Figure 5:**
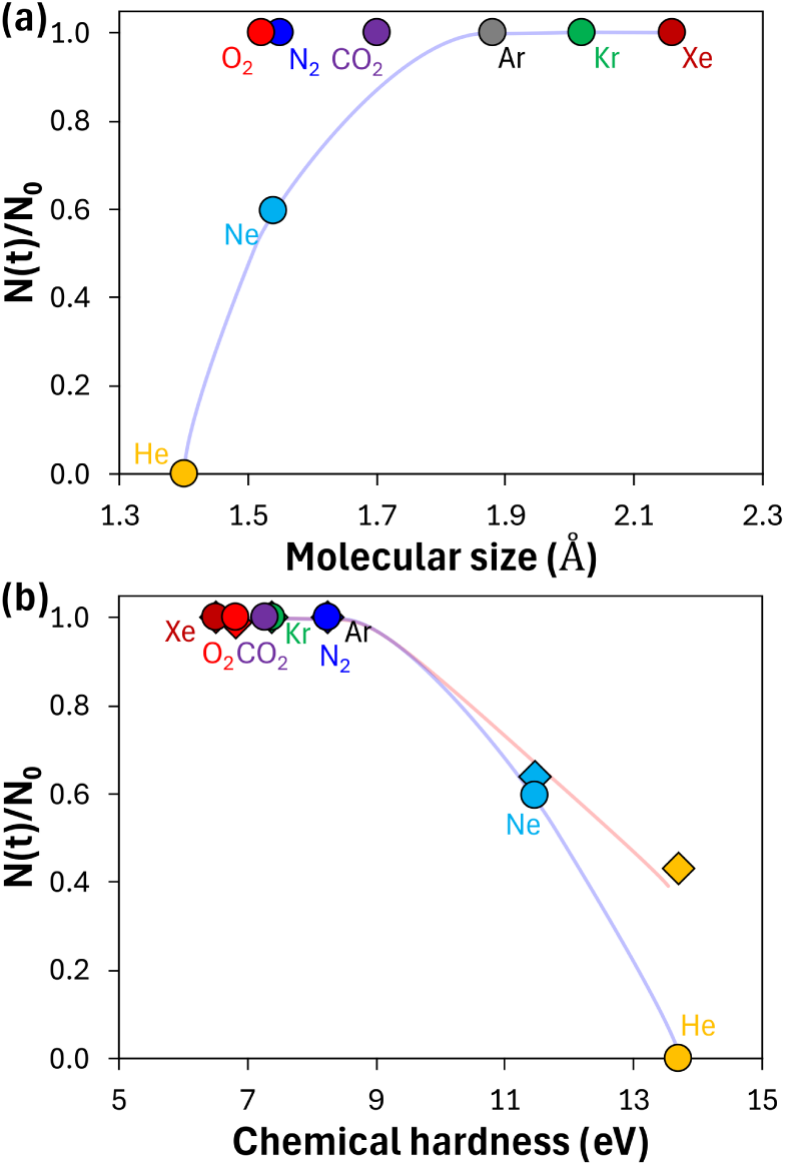
Gas fractionation behavior during bubble close-off. Relative remaining fractions, defined as *N*(*t*)/*N*_0_, of noble gases (He, Ne, Ar, Kr, and Xe) and molecular gases (N_2_, O_2_, and CO_2_) at 250 K, calculated at time when Ne reaches 60% of its initial concentration (see Section 1 of the SI for details), plotted against (a) molecular size (effective cross-sectional vdW radius during permeation) and (b) chemical hardness. For comparison, the results for He, Ne, Ar, Kr, Xe, N_2_, and CO_2_ from a previous study are shown as diamonds in (b), replotted using the chemical hardness values determined in this study.^15^ The transparent lines illustrate noble gas trends: blue lines represent our DFT-based numerical estimates, while the red line shows the trend from previous results.

In contrast, our results for molecular gases (N_2_, O_2_, and CO_2_) in Figure 5a significantly deviate from the size-based trend observed for noble gases. Previous studies reported that N_2_ and O_2_ gases appear to follow the depletion trend of noble gases by describing their molecular sizes using the collision diameters derived from hard-sphere collision theory for low-density gas systems.^13–15^ While these studies have advanced our understanding of gas transport in glacial ice, their qualitative approach has inherent limitations in capturing the intricate interfacial phenomena involved in gas permeation through structured ice matrices. Our molecular-level analysis reveals that molecular gases tend to vertically penetrate the ice layer through the hexagonal ring, making the vdW radius of the largest atom a better representation of their effective cross-sectional size (Figures 2 and 4b; see also refs 4, 16 for the diffusion of such molecular gases within the bulk ice lattice). The results for molecular gases in Figure 5a, represented by their effective cross-sectional vdW radii, deviate from the size-dependent trend established for noble gases. Despite having effective sizes smaller than those of larger noble gases or even comparable to that of Ne, these molecular gases remain almost unfractionated. This result demonstrates that molecular size alone, even when defined based on the orientation during the gas–ice interfacial penetration, may not be sufficient to explain the observed retention, highlighting the need to consider additional physicochemical factors.

Therefore, we introduced chemical hardness, which accounts for the electronic characteristics of gas molecules, as a parameter complementary to geometric factors, as illustrated in Figure 5b. We also included previously reported data for comparison.^15^ Our results in Figure 5b show that larger noble gases (Ar, Kr, and Xe) with chemical hardness values below ∼8 eV exhibit negligible depletion, a behavior attributed to both their larger atomic size and stronger adsorptive gas–ice interactions. Notably, molecular gases (N_2_, O_2_, and CO_2_) remain largely unfractionated like larger noble gases due to their relatively low chemical hardness values, in contrast to their deviation from the size-based trends in Figure 5a. This behavior arises from both steric hindrance and low chemical hardness, which enhances the adsorption affinity through increased susceptibility to intermolecular interactions. The use of chemical hardness to reconcile the fractionation patterns of both noble gases and molecular gases observed in previous studies suggests that gas–ice interfacial interactions are among the key factors governing transport phenomena. These findings underscore that molecular size alone cannot predict fractionation behavior; instead, the electronic properties governing interfacial adsorption must be considered to provide a more complete picture of gas transport through the ice layers.

Our ice layer permeation model captures the general trends in gas fractionation observed in firn air measurements, notably elucidating the mechanistic origin of the nonlinear size dependence that the previously proposed lattice size-threshold model could not fully explain.^13–15^ However, closer examination reveals non-negligible discrepancies that warrant further discussion. Most significantly, previous observations reported that when He retention reached ∼57.5%, Ne retention stood at ∼36.0% in closed-off bubbles, indicating comparable depletion rates.^15^ In contrast, our model predicts that when Ne retains 60%, He has already been completely depleted, while other gases retain most of their initial concentrations (as shown in Figure 5). This fundamental discrepancy arises from the large differences in depletion rates among gases, which result from the exponential dependence of permeation rates on effective energy barriers (see also Figure S1 in Section 1 of the SI). Furthermore, while our model qualitatively captures some aspects of the fractionation patterns, such as O_2_ depleting faster than N_2_ and Ar (consistent with O_2_ enrichment in open pores), the magnitude of O_2_/N_2_ fractionation remains minimal compared to field measurements, and the relative depletion order of N_2_ and Ar (N_2_ < Ar) differs from observation.^13–15^

These discrepancies between our DFT-based investigation and field observations suggest that bubble close-off fractionation cannot be explained solely by the ice lattice permeation, requiring the consideration of additional transport mechanisms through microstructural pathways. Reconciling our model’s large differences in depletion rates among gases (see also Figure S1 in Section 1 of the SI) with the more comparable rates observed in firn air measurements suggests the presence of alternative pathways that facilitate transport with reduced hindrance for all gas species. As the surrounding ice layer gradually thickens during the firn-to-ice transition, various structural heterogeneities likely develop that could serve as alternative pathways, including surface disorder, grain boundaries, stacking faults, and vacancy/interstitial defects.^52,63,70,72,95–98^ Among these, quasi-liquid layers along ice grain boundaries are of particular interest, as they could enable gas diffusion to proceed as if through a disordered, mobile phase even at temperatures well below the melting point.^99^

## Conclusions

In this study, we investigated the molecular-level mechanisms governing gas fractionation during bubble close-off in glacial ice. Using DFT calculations, we determined the effective permeation energy barriers for noble gases (He, Ne, Ar, Kr, and Xe) and molecular gases (N_2_, O_2_, and CO_2_) by considering both steric effects and gas–ice interfacial interactions during permeation through the ice layer. For noble gases, the permeation energy barriers are largely size-dependent and systematically increase with increasing atomic size. This trend arises from the combined effects of increased steric hindrance and stronger adsorption affinity attributed to higher polarizability. In contrast, molecular gases exhibit more complex behaviors because of their molecule-specific anisotropic electrostatic charge distributions, which cause gas–ice interactions to deviate from simple size-dependent trends. The exponential dependence of permeation rates on these energy barriers amplifies even subtle variations into substantial differences in the permeation dynamics. This fundamental principle directly accounts for the observed nonlinear depletion phenomenon, in which highly mobile He and Ne are significantly depleted, whereas gases with lower chemical hardness values (larger noble gases and molecular gases) remain largely unfractionated within closed-off bubbles due to their stronger gas–ice interactions. These findings suggest that chemical hardness, which reflects the electronic properties governing gas–ice interactions, provides crucial insights into interfacial transport phenomena.

Despite these advances, comparison with field observations reveals noteworthy complexity. The contrast between our theoretical results and measured depletion rates in firn air, particularly the discrepancy between the relatively comparable rates observed among gases and our exponentially varying predictions, indicates that natural ice systems may feature additional transport mechanisms beyond lattice permeation, such as grain boundaries potentially containing a mobile quasi-liquid phase even at low temperatures. These molecular-level insights establish a theoretical foundation for elucidating the long-term stability of gases entrapped within icy materials in both Earth’s cryosphere and icy celestial bodies, particularly for geochronological analyses such as radiometric dating with noble gas isotopes stemming from K–Ar decay and cosmic ray exposure. While not directly investigated in this study, our demonstration of the nonlinear depletion of light gases suggests potential applications in developing temperature-tunable materials for size-selective transport, offering an alternative to conventional molecular sieves, including zeolites, metal–organic frameworks, and nanoporous carbon. Future investigations should employ more sophisticated models that incorporate structural heterogeneities, such as grain boundaries and other defects, under varying thermodynamic conditions, thereby moving beyond the prevailing paradigm by integrating the rich structural complexity of natural glacial environments.

## Supplementary Material



## Notes

Analytical model and numerical estimations for gas permeation through ice layers; parameters including effective permeation energy barriers, cross-sectional van der Waals radii, chemical hardness values, and approximated attempt frequencies; temporal evolution of gas fractionation (PDF)
