# Supplementary material for: Molecular Mechanisms of Gas–Ice Interfacial Transport: Size- and Charge-Dependent Fractionation during Bubble Close-off
Source: ACS Omega. 2025 Nov 5;10(45):54700–9. doi: 10.1021/acsomega.5c08111 (PMC12631676; doi:10.1021/acsomega.5c08111)
Supplement: Supplementary file 1 [file ao5c08111_si_001.pdf]

Supporting Information:
Molecular mechanisms of gas-ice interfacial transport:
Size- and charge-dependent fractionation during bubble close-off

Yoo Soo Yi[1,*] and Yeongcheol Han[2,*]

[1] Research Institute of Basic Sciences, Seoul National University, Seoul, Korea (08826)
[2] Division of Glacier & Earth Sciences, Korea Polar Research Institute, Incheon, Korea (21990)

* Email: yys2064@snu.ac.kr
* Email: yhan@kopri.re.kr

1. Numerical estimations of bubble close-off gas fractionation in polar glaciers

Table S1. Molecular properties of noble gases and molecular gases

Figure S1. Key parameters for the numerical estimations of gas fractionation

1. Numerical estimations of bubble close-off gas fractionation in polar glaciers

This section presents an analytical model and a numerical estimation of gas permeation through the ice layer to quantitatively assess compositional fractionation during bubble close-off in polar glaciers presented in Figure 5 of the main text. The model is designed to determine the relative concentrations of noble gases (He, Ne, Ar, Kr, and Xe) and molecular gases (N_2, O_2, and CO_2) with respect to the residual fraction of Ne within closed-off bubbles.

The permeation of gas molecules from closed-off bubbles is modeled using first-order reaction kinetics as follows (see refs. 1-4 for similar diffusion models used to derive Equations S1–S8).

$$\frac{d}{dt}N(t) = -kN(t) \qquad \text{(S1)}$$

Here, $N(t)$ represents the number of gas molecules at a specific time (t), and k denotes the permeation rate constant. Integrating Equation S1 yields a time-dependent expression for $N(t)$ as follows, where N_0 is the initial number of gas molecules.

$$N(t) = N_0 \exp(-kt) \qquad \text{(S2)}$$

The diffusive flux (J) along the permeation path is expressed as follows, where P is the permeability and dC/dx is the concentration gradient.

$$J = -P\frac{dC}{dx} \qquad \text{(S3)}$$

Since our model addresses permeation through a membrane-like ice structure (a bilayer of water molecules; Figure 1), this process resembles molecular diffusion between adjacent interstitial sites. Thus, P has the same units as conventional diffusivity (m^2/s). The permeation rate (dN/dt) from the closed-off bubbles can be described as follows.

$$\frac{d}{dt}N(t) = -A \times J \qquad \text{(S4)}$$

Here, A is a scaling factor that represents the effective permeation area of the closed-off bubbles.

For systems where the external volume greatly exceeds the internal (bubble) volume, the concentration gradient can be approximated as follows, assuming that the permeated gas molecules continuously diffuse away from the ice interface.

$$\frac{dC}{dx} \approx \frac{C_{\mathrm{in}} - C_{\mathrm{out}}}{L} \approx \frac{C_{\mathrm{in}}}{L} = \frac{N(t)}{LV_{\mathrm{b}}} \qquad \text{(S5)}$$

Here, C_{in} and C_{out} refer to the gas concentrations inside and outside the closed-off bubble, respectively. L is the ice layer thickness; and V_{b} represents the volume of the closed-off bubble. By substituting Equations S3 and S5 into S4, the temporal evolution of gas molecules within closed-off bubbles can be expressed as follows.

$$\frac{d}{dt}N(t) = -AP\frac{N(t)}{LV_{\text{b}}} \qquad \text{(S6)}$$

Given the similarity to molecular diffusion between adjacent interstitial sites, the permeability (P) follows an Arrhenius-type relationship as shown below, where E_{P} is the effective permeation energy barrier, T is the temperature, k_{B} is the Boltzmann constant, and P_0 is the preexponential factor.

$$P = P_0 \exp\left(-\frac{E_{\text{P}}}{k_{\text{B}}T}\right) \qquad \text{(S7)}$$

By combining Equations S1, S6, and S7, the permeation rate constant (k) becomes as follows.

$$k = \frac{AP}{LV_{\text{b}}} = \frac{AP_0}{LV_{\text{b}}} \times \exp\left(-\frac{E_{\text{P}}}{k_{\text{B}}T}\right) \qquad \text{(S8)}$$

For numerical estimations, we used E_{P} values from our DFT calculations (Table S1), an ice layer thickness of $L \approx 10$ Å (1.0×10^{-9} m), and a bubble radius of $r \approx 1$ μm (1.0×10^{-6} m), yielding a volume of $V_{\text{b}} \approx 4.19 \times 10^{-18}$ m^3 [$= (4/3)\pi r^3$]. Table S1 provides the E_{P}, cross-sectional van der Waals (vdW) radius, and chemical hardness for each gas species. To establish the relative trends of the permeation rate constants (k) for other gases with respect to Ne, the scaling factor (A) is determined by using the E_{P} of Ne, in accordance with Equations S2 and S8. This scaling factor (A), i.e., the effective permeation area, is then consistently applied to all other gas species. By rearranging these equations, the scaling factor (A) can be calculated as follows.

$$A = \frac{-\ln(0.6)}{t_{\text{c}}} \times \frac{LV_{\text{b}}}{P_0(\text{Ne})} \times \exp\left[\frac{E_{\text{P}}(\text{Ne})}{k_{\text{B}}T}\right] \qquad \text{(S9)}$$

In this equation, the timescale t_{c} is set as the time required for the remaining fraction of Ne to reach 60%. This condition facilitates straightforward comparisons of the relative depletion timescales for other gases.

Atmospheric gases trapped in polar snowpack typically require over ~100 years to become encapsulated within closed-off bubbles, as evidenced by Devon Island data (bubble close-off depth of ~59 m, average firn density of ~0.6 g/cm^3, mean snow accumulation rate of 0.276 m H_2O/yr).[5] However, the precise timescale for close-off fractionation in polar glaciers is not well constrained. To facilitate numerical comparisons, we define the characteristic time

(t_c) as the time required for Ne to reach 60% of its initial concentration. We set t_c to 1 second (yielding $A \approx 2.01 \times 10^{-11}$ m^2). This choice serves as a normalization factor and does not imply that actual Ne depletion occurs on this timescale. Rather, this normalized timescale ($\tau = t/t_c$) allows a direct comparison of the relative permeation rates of different gases.

The preexponential factor (P_0) in Equation S7 is the product of a proportional coefficient (α_P) and an attempt frequency (ν_P): $P_0 = \alpha_\mathrm{P} \times \nu_\mathrm{P}$. A more rigorous estimation of ν_P could be achieved by using Vineyard's equation, which establishes the relationship between ν_P and the ratio of vibrational partition functions at the initial and transition states (see refs. 2, 6-8). However, such an approach would require extensive and computationally demanding phonon calculations for both the initial and transition states. Therefore, in this study, we adopt a simplified model to estimate the relative trends in the attempt frequencies based on the molecular mass and potential well depth.

This model assumes that a gas molecule's attempt frequency to reach the transition state is governed by the depth and curvature of the potential well, whereas the probability of successful permeation is dictated by the height of the effective permeation energy barrier. Although structural rearrangement during the IM1-to-TS transition influences rigorous attempt frequency calculations using Vineyard's equation, our simplified model assumes these structural variations have minimal impact on comparative permeation rates among gas species. In our model, the vibrational motion of a permeating gas molecule is determined by its molecular mass (m) and the potential well in which it is trapped on the ice layer. The depth of this potential well (ϵ) can be approximated by the magnitude of $E(\mathrm{IM1}) - E(\mathrm{IS})$ (Figure 4a). Assuming a simple model like the Lennard–Jones potential, we anticipate the relationship $\nu_\mathrm{P} \propto \sqrt{\epsilon/m}$. We adopt an attempt frequency for He of 1.0×10^{12} Hz (s^{-1}), which is consistent with the typical range of solid-state phonon frequencies. The relative attempt frequencies (ν_P) for other gases are estimated using the above relationship and range from 5.09×10^{11} Hz (Xe) to 1.18×10^{12} Hz (O_2), as shown in Figure S1a. The nonmonotonic trend for noble gases (He > Ne < Ar > Kr > Xe) results from the interplay between ϵ and m. While the actual potential landscape is likely more complex, this approximation provides a reasonable assessment of relative trends.

The proportional coefficient (α_P) can be approximated according to similar molecular diffusion models (details in refs. 2, 6-8) as follows.

$$\alpha_\mathrm{P} = \frac{n_\mathrm{p} d_0^2}{2 n_\mathrm{d}} \approx \frac{d_0^2}{2} \qquad \text{(S10)}$$

In our model, permeation occurs along a single ($n_\mathrm{p} = 1$), 1-dimensional ($n_\mathrm{d} = 1$) path (as shown in Figure 1) with a characteristic distance (d_0) of ~10 Å (resulting in $\alpha_\mathrm{P} \approx 5.0 \times 10^{-19}$

m^2). While the gas-specific $P_0(\text{gas})$ may vary among gases, the overall permeability (P) remains governed primarily by its exceptional dependence on E_P and T, as evidenced by Equation S7.

The permeation rate constant (k) for each gas species can be formulated as follows.

$$k(\text{gas}) = \frac{-\ln(0.6)}{t_\text{c}} \times \frac{P(\text{gas})}{P(\text{Ne})} \qquad (\text{S11})$$

Due to the exponential dependence on E_P (Equation S7), gases other than He and Ne (larger noble gases and molecular gases) exhibit permeation rate constants (k) 6 to 28 orders of magnitude lower than Ne. Consequently, we obtain the temporal evolution of the normalized gas concentrations (Equations S2 and S11) as presented in Figure S1b.

Table S1. Molecular properties of noble gases (He, Ne, Ar, Kr, and Xe) and molecular gases (N_2, O_2, and CO_2). The parameters include the effective cross-sectional van der Waals radius (molecular size), chemical hardness (η) derived from NIST data using the ionization potential and electron affinity (see refs. 9-12), DFT-based chemical hardness (η_{DFT}) determined in this study by varying the number of electrons for each molecule, and effective permeation energy barrier (E_{P}) determined in this study (see Figure 4).

Gas species	Molecular size (Å)	η (eV)	η_{DFT} (eV)	E_{P} (eV)
		**Noble gases**		
He	1.40	12.40	13.69	0.18
Ne	1.54	10.80	11.47	0.47
Ar	1.88	7.90	8.24	1.22
Kr	2.02	7.00	7.37	1.55
Xe	2.16	6.10	6.50	1.83
		**Molecular gases**		
N_2	1.55	7.80	8.23	0.94
O_2	1.52	5.80	6.82	0.76
CO_2	1.70	7.20	7.27	0.89

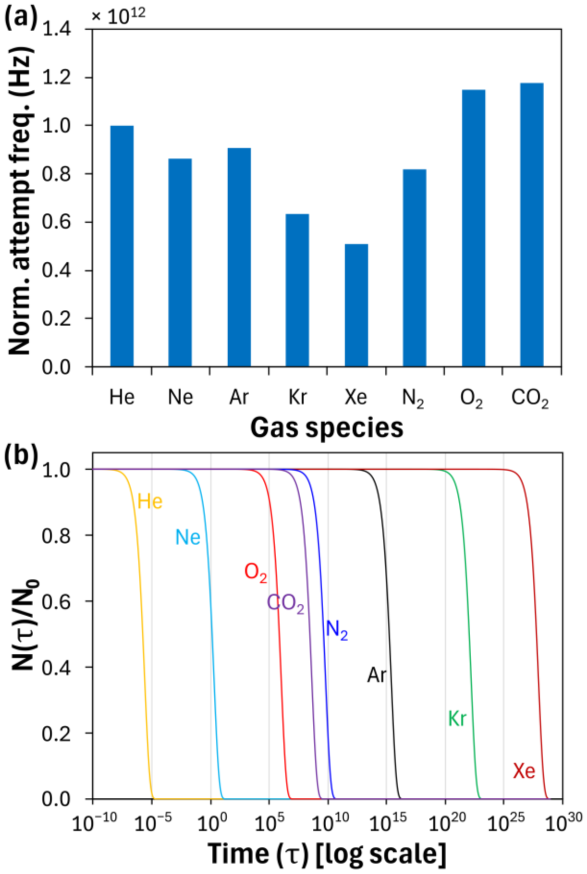

Figure S1. Key parameters for the numerical estimation of gas fractionation. (a) Attempt frequencies (ν_{P}) for each gas, normalized to the value for He. The attempt frequencies were approximated according to the assumptions outlined in Section 1 of the SI. (b) Temporal evolution of the remaining gas fraction, defined as $N(\tau)/N_0$, plotted against a normalized timescale ($\tau = t/t_{\mathrm{c}}$), where t_{c} is the time at which the remaining fraction of Ne reaches 60% of its initial value.

REFERENCES

(1) Ikeda-Fukazawa, T.; Fukumizu, K.; Kawamura, K.; Aoki, S.; Nakazawa, T.; Hondoh, T. Effects of molecular diffusion on trapped gas composition in polar ice cores. *Earth Planet. Sci. Lett.* **2005**, *229* (3), 183–192. DOI: 10.1016/j.epsl.2004.11.011.

(2) Koettgen, J.; Zacherle, T.; Grieshammer, S.; Martin, M. Ab initio calculation of the attempt frequency of oxygen diffusion in pure and samarium doped ceria. *Phys. Chem. Chem. Phys.* **2017**, *19* (15), 9957–9973. DOI: 10.1039/C6CP04802A.

(3) de Koker, N.; Stixrude, L. Theoretical computation of diffusion in minerals and melts. *Rev. Mineral. Geochem.* **2010**, *72* (1), 971–996. DOI: 10.2138/rmg.2010.72.22.

(4) Maghami, S.; Mehrabani-Zeinabad, A.; Sadeghi, M.; Sánchez-Laínez, J.; Zornoza, B.; Téllez, C.; Coronas, J. Mathematical modeling of temperature and pressure effects on permeability, diffusivity and solubility in polymeric and mixed matrix membranes. *Chem. Eng. Sci.* **2019**, *205*, 58–73. DOI: 10.1016/j.ces.2019.04.037.

(5) Huber, C.; Beyerle, U.; Leuenberger, M.; Schwander, J.; Kipfer, R.; Spahni, R.; Severinghaus, J. P.; Weiler, K. Evidence for molecular size dependent gas fractionation in firn air derived from noble gases, oxygen, and nitrogen measurements. *Earth Planet. Sci. Lett.* **2006**, *243* (1), 61–73. DOI: 10.1016/j.epsl.2005.12.036.

(6) Yi, Y. S.; Han, Y. Theoretical insights into gas migration within ice on earth and icy celestial bodies. *ACS Earth Space Chem.* **2024**. DOI: 10.1021/acsearthspacechem.4c00266.

(7) Kong, L.; Lewis, L. J. Surface diffusion coefficients: Substrate dynamics matters. *Phys. Rev. B* **2008**, *77* (16), 165422. DOI: 10.1103/PhysRevB.77.165422.

(8) Kong, L. T.; Lewis, L. J. Transition state theory of the preexponential factors for self-diffusion on Cu, Ag, and Ni surfaces. *Phys. Rev. B* **2006**, *74* (7), 073412. DOI: 10.1103/PhysRevB.74.073412.

(9) Reed, J. L. Electronegativity: Chemical Hardness I. *J. Phys. Chem. A* **1997**, *101* (40), 7396–7400. DOI: 10.1021/jp9711050.

(10) Chakraborty, D.; Chattaraj, P. K. Conceptual density functional theory based electronic structure principles. *Chem. Sci.* **2021**, *12* (18), 6264–6279. DOI: 10.1039/D0SC07017C.

(11) Geerlings, P.; De Proft, F.; Langenaeker, W. Conceptual Density Functional Theory. *Chem. Rev.* **2003**, *103* (5), 1793–1874. DOI: 10.1021/cr990029p.

(12) Linstrom, P. J.; Mallard, W. G., Eds. NIST Chemistry WebBook, NIST Standard Reference Database Number 69. National Institute of Standards and Technology. Gaithersburg, MD. 2025. https://webbook.nist.gov/chemistry (accessed 2025–08–12).
